# Editorial: Endophytic fungi: secondary metabolites and plant biotic and abiotic stress management

**DOI:** 10.3389/fmicb.2024.1345210

**Published:** 2024-01-29

**Authors:** Mina Salehi, Naser Safaie

**Affiliations:** ^1^Department of Biotechnology and Plant Breeding, Faculty of Agriculture, Tarbiat Modares University, Tehran, Iran; ^2^Department of Plant Pathology, Faculty of Agriculture, Tarbiat Modares University, Tehran, Iran

**Keywords:** endophytism, mycorrhizae, bioactive compounds, biological control, sustainable agriculture

Endophytes can live inside plant tissues without causing visible symptoms in their hosts (Hardoim et al., 2015). Endophytic fungi have gained remarkable attention in research, as they not only provide novel sources of bioactive secondary metabolites (SMs) that are the backbone of many drugs but can also protect the host plant from biotic and abiotic stresses that pose a serious threat to crop food safety and security. Hence, endophytic fungi have had a considerable impact on medicine, agriculture, and industry, and thus the economy. Previous studies (Torkamani et al., 2014; Tashackori et al., 2018; Salehi et al., 2019) presented the significant potential of fungal elicitors as well as co-cultivation of the endophytic fungus and plant cells for paclitaxel biosynthesis increment in a *Corylus avellana* cell culture. In this Research Topic, Zhang et al. showed that roots inoculated with endophytes promoted the production of polyphyllin, an antiviral, analgesic, antibacterial, and anti-inflammatory agent, in *Paris polyphylla* rhizomes, likely by the upregulation of downstream cytochrome P450 and UDP-glycosyltransferase genes involved in polyphyllin biosynthesis. Santra and Banerjee described endophytic *Curvularia eragrostidis* as a potent anti-microbial producer. This isolate produced volatile organic compounds (VOCs) that can be used as a tool for sustainable agriculture by preventing the growth of dangerous phytopathogens. In addition, bioactive metabolites produced by *Curvularia eragrostidis* can be a powerful alternative to traditional antibiotics and effectively curb the deadly diseases caused by multidrug-resistant gram-positive and gram-negative bacterial pathogens in the human population. Furthermore, numerous investigations have demonstrated that the majority of *Trichoderma* spp. can biosynthesize bioactive compounds and display antagonistic effects against nematodes and fungi that cause plant disease (Yao et al.). These bioactive compounds, which include cell wall-degrading enzymes and secondary metabolites, can effectively decrease plant disease, promote crop resistance, and enhance plant growth (Yao et al.). Gangaraj et al. showed that *Aspergillus niger* produced different antimicrobial metabolites and displayed high potential for the biocontrol of soil-borne diseases including guava wilt and others. Several endophytic fungi have been isolated from *Brassica oleracea*, showing different advantages for the host, including cold tolerance, plant growth promotion, and induction of resistance to pests (*Mamestra brassicae*) and pathogens (*Xanthomonas campestris*; Poveda et al.). These endophytic fungi improved the growth of *B. carinata, B. rapa, B. nigra, B. napus*, and *B. juncea*, likely because of auxin and siderophore biosynthesis as well as P solubilization, or cellulase, amylase, or xylanase activity (Poveda et al.). SMs produced by endophytic fungi include phenols, alkaloids, polyketides, quinones, steroids, enzymes, and peptides, which display diverse biological activities such as anticancer, insecticidal, antioxidant, cytotoxic, antibacterial, antiviral, antifungal, and antimalarial properties (Hardoim et al., 2015). A microbial fermentation process would be the most favorable means for the production of bioactive secondary metabolites. Microorganisms are fast-growing, and their genetic manipulation is often relatively easy and can be scaled up to an industrial level (Salehi et al., 2018). The endophytic fungi of *Alisma orientale* produced numerous secondary metabolites with strong antibacterial and antioxidant activities (Shen et al.). Abo-Kadoum et al. reported that endophytic fungi in different plant species can offer an incredible way to supply a steady yield of resveratrol, a strong contender to treat a wide range of human diseases. Mulberry branch extracts promoted the mycelial growth and biosynthesis of bioactive compounds of *Sanghuangporus vaninii*, widely used in traditional medicine, by upregulating hydrolase family genes (Huo et al.).

Mycoendophyte “*Trichoderma* sp.” displayed antioxidant, antifungal, and a-glucosidase inhibitory activities and also greatly inhibited the growth of plant pathogens including *Aspergillus niger, Fusarium* sp., and *Trichoderma viride* (Elfiati et al.). Ebrahimi et al. screened two effective endophytic fungal isolates, *Coniochaeta endophytica* and *Chaetomium globosum*, for the biocontrol of apple scab disease caused by *Venturia inaequalis*. Chemical control of plant disease can be destructive to the environment, and repeated use of chemical fungicides can lead to the prevalence of agrochemical-resistant strains. The application of endophytic fungi for enhancing tolerance or resistance to biotic stress is a greener alternative approach that could reduce the use of chemicals in agriculture, conferring benefits to both the environment and human health (Bhardwaj et al.). Using endophytic fungi for the protection of plants against abiotic stresses could additionally mitigate the detrimental effect of global climate change on agriculture. Under greenhouse conditions, the detrimental effects of salinity and drought stresses on barley were lessened by halotolerant endophytic fungi including *Neocamarosporium goegapense, N. chichastianum*, and *Periconia macrospinosa*, which were isolated from the roots of salt lake plants growing in the central desert of Iran. Indeed, such endophytic fungi have evolved various strategies to survive in harsh desert environments and are essential in providing their host plants with resistance against extreme environmental stress (Hosseyni Moghaddam et al.).

Mycorrhizal fungi are the other major class of endophytic fungi. They inhabit roots but spread out into the rhizosphere. Mycorrhizae can also enhance plant tolerance against different abiotic stresses related to global climate change, including heat, metals, salinity, drought, and extreme temperatures. Therefore, they can help plants to cope with the changing climate (Bonfante and Genre, 2010). Additionally, Zhao et al. reported that the inoculation of *Alkanna tinctoria* using arbuscular mycorrhizal *Rhizophagus irregularis* enhanced the production of shikonin and alkannin.

## Author contributions

MS: Writing—original draft, Writing—review & editing. NS: Writing—original draft, Writing—review & editing.

## References

[B1] Bonfante P. Genre A. (2010). Mechanisms underlying beneficial plant–fungus interactions in mycorrhizal symbiosis. Nat. Commun. 1, 48. 10.1038/ncomms1046 20975705

[B2] Hardoim P. R. Van Overbeek L. S. Berg G. Pirttil,ä A. M. Compant S. Campisano A.. (2015). The hidden world within plants: ecological and evolutionary considerations for defining functioning of microbial endophytes. Microbiol. Mol. Biol. Rev. 79, 293–320. 10.1128/MMBR.00050-14 26136581 PMC4488371

[B3] Salehi M. Moieni A. Safaie N. (2018). Elicitors derived from hazel (*Corylus avellana* L.) cell suspension culture enhance growth and paclitaxel production of *Epicoccum nigrum*. Sci. Rep. 8, 12053. 10.1038/s41598-018-29762-3 30104672 PMC6089963

[B4] Salehi M. Moieni A. Safaie N. Farhadi S. (2019). New synergistic co-culture of *Corylus avellana* cells and *Epicoccum nigrum* for paclitaxel production. J. Indust. Microbiol. Biotechnol. 46, 613–623. 10.1007/s10295-019-02148-8 30783891

[B5] Tashackori H. Sharifi M. Chashmi N. A. Behmanesh M. Safaie N. (2018). Piriformospora indica cell wall modulates gene expression and metabolite profile in Linum album hairy roots. Planta 248, 1289–1306. 10.1007/s00425-018-2973-z 30109409

[B6] Torkamani M. R. D. Jafari M. Abbaspour N. Heidary R. Safaie N. (2014). Enhanced production of valerenic acid in hairy root culture of Valeriana officinalis by elicitation. Cent. Eur. J. Biol. 9, 853–863. 10.2478/s11535-014-0320-3

